# The role of iron metabolism in chronic diseases related to obesity

**DOI:** 10.1186/s10020-022-00558-6

**Published:** 2022-11-05

**Authors:** Fangyi Qiu, Lei Wu, Guang Yang, Cong Zhang, Xiaofang Liu, Xiance Sun, Xin Chen, Ningning Wang

**Affiliations:** 1 School of Public Health, Dalian Medical University, Dalian, Liaoning People’s Republic of China; 2 Department of Nutrition and Food Hygiene, Global Health Research Center, School of Public Health, Dalian Medical University, Dalian, Liaoning People’s Republic of China; 3 Occupational and Environmental Health Department, Global Health Research Center, School of Public Health, Dalian Medical University, Dalian, People’s Republic of China; 4 Department of Epidemiology, School of Public Health, Dalian Medical University, Dalian, Liaoning People’s Republic of China

**Keywords:** Iron metabolism, Obesity, Chronic diseases, Ferroptosis

## Abstract

Obesity is one of the major public health problems threatening the world, as well as a potential risk factor for chronic metabolic diseases. There is growing evidence that iron metabolism is altered in obese people, however, the highly refined regulation of iron metabolism in obesity and obesity-related complications is still being investigated. Iron accumulation can affect the body’s sensitivity to insulin, Type 2 diabetes, liver disease and cardiovascular disease. This review summarized the changes and potential mechanisms of iron metabolism in several chronic diseases related to obesity, providing new clues for future research.

## Introduction

Iron is a metal element abundant on Earth and is involved in the composition of all living organisms because of its role in various metabolic processes, including oxygen transport, DNA synthesis, and electron transport (Abbaspour et al. 2014). Dietary iron is transported in the small intestine or ileum, which is mediated by the iron transporter divalent metal transporter 1 (DMT1) (Gulec et al. 2014), and excess iron in the body is stored in the form of ferritin, mainly in the liver, spleen, bone marrow, and small intestine mucosa. Generally, the transferrin-ferritin axis is responsible for maintaining iron homeostasis in the body. Hepcidin is a peptide hormone secreted by the liver that binds to and promotes the internalization and degradation of Ferroportin (Fpn), resulting in a diminished export of non-heme iron from tissues to the circulatory system (Nemeth et al. 2004). The decreasing of hepcidin prompts an overburden of iron in plasma, while the overproduction of hepcidin leads to hypoferremia and the anemia of inflammation (Camaschella et al. 2020). Under physiological circumstances, the intricate and exact iron homeostasis system guarantees iron fixations in cells and keeps intracellular iron overload (Ganz 2013). The Fenton reaction occurs when a large amount of iron aggregates, which H_2_O_2_ generates strong oxidizing power of hydroxyl radicals (–OH) in the presence of Fe^2+^, and triggers more reactive oxygen species (ROS). The occurrence of the Fenton reaction and the significant amount of reactive oxygen species production induce ferroptosis, which is an iron-dependent form of programmed cell death newly discovered in 2012 (Dixon et al. 2012).

**Fig. 1:**
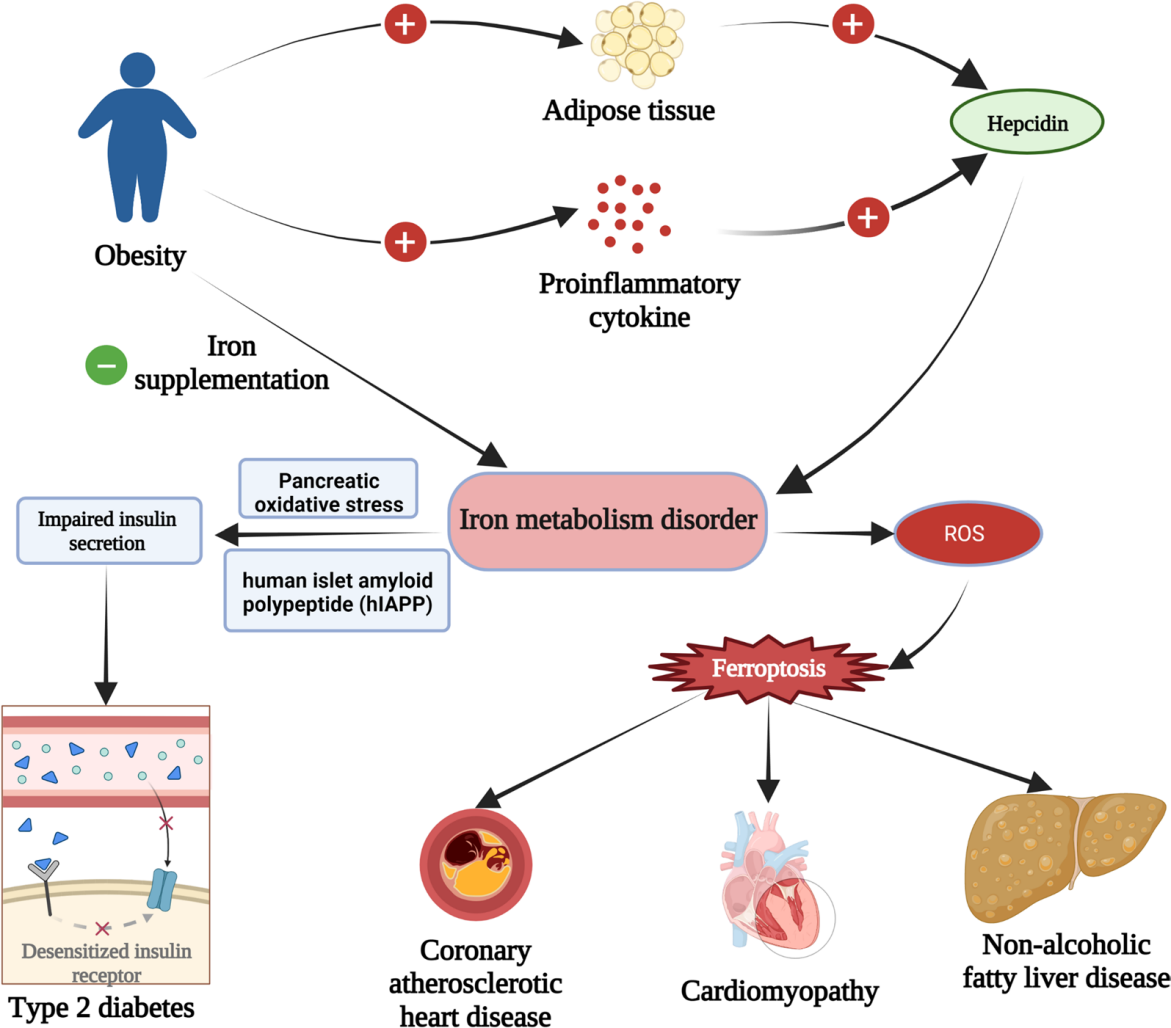
Overview of the iron metabolism in obesity and related chronic diseases. Obesity leads to systemic iron deficiency and tissue iron overload, causing non-alcoholic fatty liver disease, Type 2 diabetes, coronary atherosclerotic heart disease, and cardiomyopathy

Type 2 diabetes, insulin resistance, non-alcoholic fatty liver disease (NAFLD), hypertension, and atherosclerosis are all directly linked to the development of obesity. Additionally, it has shown that these metabolic disorders are accompanied by variations in iron. In recent decades, obese adults are found to be more likely to have a low iron status than non-obese ones, despite getting adequate iron intake (Lecube et al. 2006). In particular, obese patients with combined chronic inflammatory conditions are more susceptible to hypoferritinemia (serum ferritin deficiency), which can be considered to be related to iron deficiency caused by the inflammatory response (Yanoff et al. 2007). Serum ferritin levels are positively correlated with serum insulin and HOMA-IR values (Moore Heslin et al. 2021), and there is evidence of an association between serum iron levels and metabolic syndrome (Sachinidis et al. 2020). Lowering plasma ferritin was proved to improve NAFLD in obese patients, suggesting that consideration of iron status is imperative in the treatment of obesity-related metabolic dysfunction (Moore Heslin et al. 2021). Here, we summarize the pathophysiological mechanisms of iron action in obesity and its related metabolic diseases to provide new perspectives for the prevention and treatment of obesity (Fig. 1).

## The influence of iron metabolism disorder to health

Iron deficiency is one of the most common nutritional deficiencies in the world, affecting more than 2 billion people (Denic and Agarwal 2007), and is currently the leading cause of common nutritional anemia. People with iron deficiency have a loss of appetite, which can be restored by iron supplementation. A previous study in Kenya found a link between iron deficiency and decreased appetite in primary school-aged children and demonstrated that iron supplementation improved growth and appetite (Lawless et al. 1994). Moreover, it has been suggested that dietary iron level plays an essential role in the manipulation of appetite and metabolism through cAMP-responsive element binding protein (CREB)-dependent regulation of leptin expression, a hormone that is secreted primarily by adipose tissue and is responsible for regulating feeding behavior(Gao et al. 2015). In addition, iron deficiency impairs adaptive thermogenesis, which is the part of our body’s defense mechanism against external stimulation (e.g., excess energy intake and cold temperature), thereby exacerbating obesity and metabolic dysfunction (Yook et al. 2021).

In humans, iron overload is considered when the transferrin saturation is higher than 45% in females and 50% in males (Tanno and Miller 2010). When serum transferrin saturation exceeds 60%, non-transferrin-bound iron (NTBI) accumulates in the circulation and causes cellular damage (Lal 2020). Iron can accumulate in multiple organs, most commonly in the liver, heart and pancreas (Pietrangelo 2010). In addition, sex hormones may play an extensive role in iron metabolism. Epidemiological studies have found a two–threefold increase in serum ferritin levels in premenopausal versus postmenopausal women (Yang et al. 2012). Mean serum iron levels were significantly higher in participants using contraception (Fischer et al. 2021). Hou et al. (2012) found the presence of estrogen response element (ERE) in the hepcidin gene promoter, whose expression is regulated by estrogen. As a result, menopausal women are more likely to suffer from iron overload-related diseases. Iron overload will increase the risk of liver fibrosis and cirrhosis, hepatocellular carcinoma, cardiomyopathy, arthritis and diabetes and some of that will be described in detail later.

## Iron metabolism and obesity

**Fig. 2:**
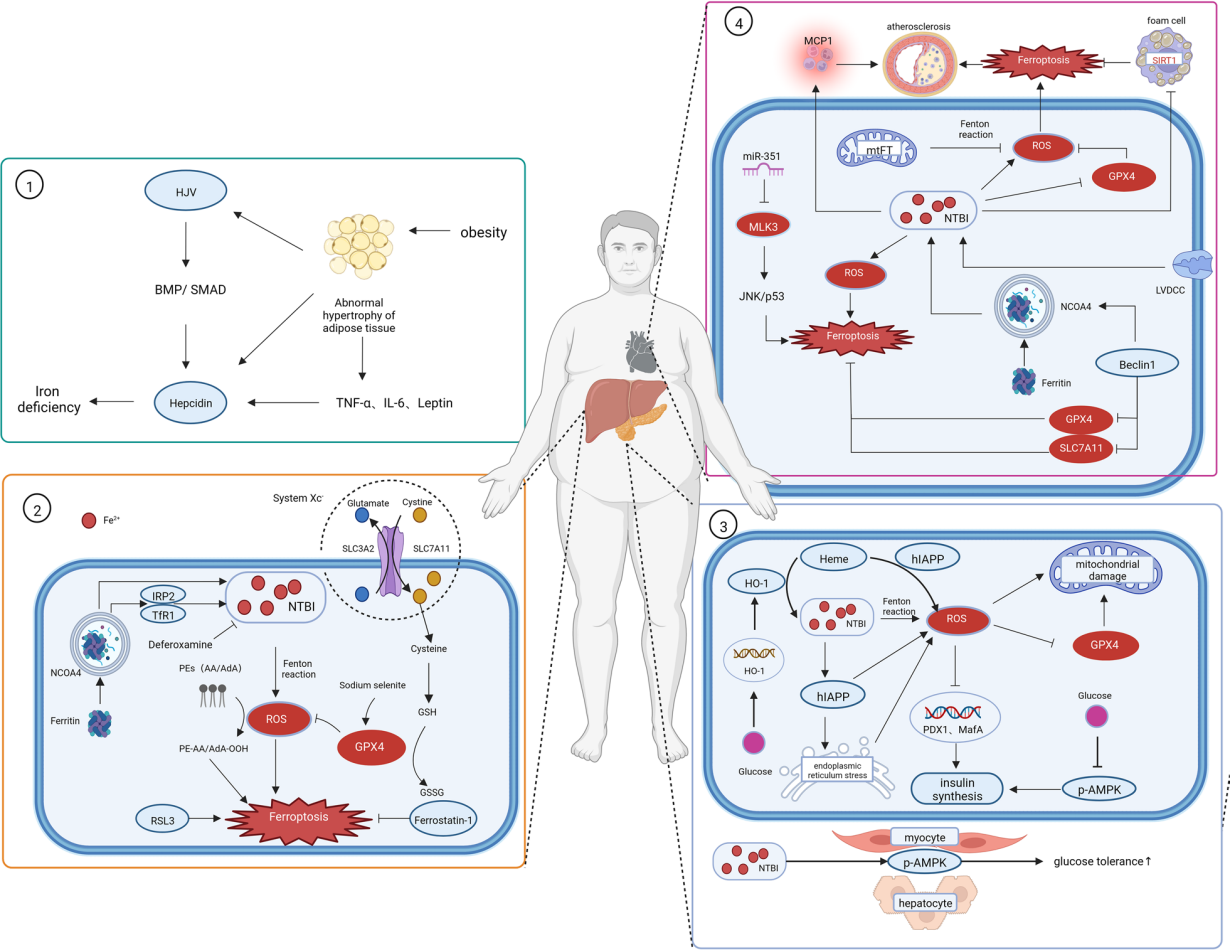
Schematic diagram of the relationship between iron metabolism disorder and obesity and related metabolic diseases. Hepcidin over-expression can induce iron deficiency, and hemojuvelin (HJV) can promote hepcidin expression through the BMP/SMAD signaling pathway. ① In obese individuals, adipose tissue can regulate expression of hepcidin and HJV through overproduction of pro-inflammatory adipokines including IL-6, TNF-α and leptin; meanwhile, it also directly expresses hepcidin and HJV at high levels, which contribute to iron deficiency in obese individuals. ② The role of iron overload and ferroptosis in nonalcoholic fatty liver disease (NAFLD). Nuclear receptor coactivator (NOCA)4 contributes to iron overload by translocating ferritin to lysosomes and increasing the expression of iron regulatory protein (IRP)2 and transferrin receptor (TfR). Lipid peroxidation of membrane phospholipids can be eliminated by parallel metabolic pathways, including the cyst(e)ine/GSH/GPX4 axis, as well as the ferroptosis inducer (RSL3). In addition, several ferroptosis inhibitors such as sodium selenite (GPX4 activator), deferoxamine (iron-chelating agent) and ferrostatin-1 (ferroptosis inhibitor) can alleviate the onset and progression of NAFLD. ③ Iron-mediated toxic effects in beta-cells. Iron-mediated beta-cell toxicity is mainly due to reactive oxygen species (ROS) accumulation through the Fenton reaction. Excess ROS causes mitochondrial damage, leading to defects in the synthesis and secretion of insulin. ROS also influences the activity of the PDX1, MafA and AMPK, critical transcription factors for the control of insulin gene expression. In turn, hyperglycemia increases heme oxygenase (HO)-1 gene expression, exacerbates iron overload, promotes oxidative stress and the development of T2DM. In addition, misfolding and aggregated deposition of human islet amyloid polypeptide (hIAPP) due to iron overload can also lead to oxidative stress through endoplasmic reticulum stress, mitochondrial damage and complex formation by binding to heme. Besides, iron overload can stimulate glucose uptake and fatty acid oxidation by activating AMPK phosphorylation in skeletal muscle and liver, leading to an increase in glucose tolerance. ④ The relationship between iron overload and cardiovascular disease. Accumulation of iron in the heart has been supposed to depend on the penetration of Fe^2+^ through the L-type voltage-dependent Ca^2+^ channel (LVDCC). Non-transferrin-bound iron (NTBI) promotes atherogenesis by leading to ROS production through the Fenton reaction and stimulating monocyte chemotactic protein (MCP)1-mediated monocyte aggregation. In addition, iron overload inhibits SIRT1 and glutathione peroxidase (GPX)4, contributing to ferroptosis in foam cells and thus leading to atherosclerosis. Moreover, mixed lineage kinase (MLK)3 and Beclin1 can induce ferroptosis in cardiomyocytes through JNK/p53, and by affecting the levels of NOCA4, SLC7A11 and GPX4, respectively.

As mentioned earlier, studies have linked obesity to iron deficiency (Zhao et al. 2015), and hepcidin, as the central regulator of iron metabolism, may be a potential mediating factor. On the one hand, adipose tissue of obese individuals changes in morphology and function and secretes some proinflammatory adipokines which can stimulate the expression of hepcidin. On the other hand, adipose tissue of obese individuals can also directly express hepcidin and hemojuvelin (HJV) (Fig. 2 ①).

### Hepcidin and HJV in obesity

Hepcidin is a peptide hormone of a single hairpin structure that is composed of 8 cysteine residues with four disulfide bonds. Its amino acid sequences are highly conserved in mammals (Mead et al. 2019). Hepcidin was first discovered as an antimicrobial peptide that is primarily involved in the body’s immune response (Park et al. 2001). The regulation of hepcidin in the inflammatory response is mainly mediated through the IL-6/STAT (signal transducers and activators of transcription) signaling pathways (Verga Falzacappa et al. 2007). Hepcidin is mainly synthesized in the liver and is also secreted by adipocytes and macrophages (Park et al. 2001; Wozniak et al. 2009). Previous studies in mice have found conflicting results regarding the relationship between strain, sex, and hepcidin levels (McLachlan et al. 2017). Unlike humans, which have only one hepcidin gene, mice have two: hepcidin-1 (Hamp1) and hepcidin-2 (Hamp2), which share 68% similarity at the protein level but differ in function (Ilyin et al. 2003; Nicolas et al. 2001). Only the role of hepcidin-1 in iron metabolism has been confirmed (Lou et al. 2004). McLachlan’s study showed that both sex and strain have significant effects on Hamp1 expression levels, confirming previous findings, whereas the sex-by-strain interaction was suggestively significant (McLachlan et al. 2017). Excess secretion of hepcidin due to inflammation can result in iron deficiency, while inadequate secretion of hepcidin can lead to iron overload. It makes sense that intervention of lexaptepid, an antagonist of hepcidin, ameliorates the decrease in the serum iron level caused by lipopolysaccharide (LPS)-induced systemic inflammation (van Eijk et al. 2014). In addition, a population-based study has found that individuals with a high body mass index (BMI) gain a significant elevation in the serum hepcidin expression level compared with those with a low BMI (Vuppalanchi et al. 2014). In rodent research, a high-fat diet (HFD, 60% energy from fat) caused increased expression of hepcidin in visceral adipose tissue of mice, as well as a significant increase in interleukin (IL)-6 at both mRNA and protein levels (Gotardo et al. 2013). Heme oxygenase (HO-1) can inhibit the expression of hepcidin and decrease the expression of IL-6 and tumor necrosis factor (TNF)α in the liver of obese mice (Puri et al. 2017). Collectively, increased expression of hepcidin due to chronic inflammation in obesity may be one of the important incentives of iron deficiency in obese individuals.

HJV, also known as repulsive guidance molecule C (RGMc), is a glycoprotein that is mainly expressed in skeletal muscle, heart, liver and adipocyte (Luciani et al. 2011). Similar to hepcidin in function, RGMc is also involved in innate immune response (Wu et al. 2017). It has been suggested that HJV is necessary for inflammation-induced hepcidin expression (Canali et al. 2017). HJV deficiency severely inhibits the BMP6/SMAD(Bone morphogenetic protein and Drosophila mothers against decapentaplegic) signaling pathway, thereby disrupting the synergistic effects of the BMP6/SMAD and IL-6/STAT signaling pathways and inhibiting inflammation-induced expression of hepcidin (Fillebeen et al. 2018). In addition, antibodies to HJV can effectively inhibit the increase of hepcidin expression level in both inflammatory and non-inflammatory status and increase the Hb level (Kovac et al. 2016). Notably, HJV is also expressed in adipocytes at mRNA and protein levels, and its mRNA expression was highly increased in adipose tissue from obese individuals and positively correlated with hepcidin expression levels (Luciani et al. 2011). More importantly, HJV produced by adipose tissue seems to be biologically active, as cultured adipocytes increased their hepcidin expression via BMP pathway. Meanwhile, blood concentration of soluble HJV was significantly increased in obese patients compared to controls, suggesting that the adipose tissue may have a role in iron homeostasis in obesity and in erythropoiesis through the action of HJV (Luciani et al. 2011). Therefore, abnormal expression of HJV in adipose tissues of obese individuals may cause iron deficiency through exaggerating hepcidin expression.

### Adipokines and proinflammatory cytokines and iron metabolism in obesity

Adipokines can coordinate hepcidin in the liver. In addition to adipokines such as leptin, M1-type macrophages recruited by hypertrophy or dysfunctional adipose tissues in obesity further secrete proinflammatory cytokines including TNFα and IL-6, which can regulate hepcidin expression (Cao 2014).

Among inflammatory cytokines secreted by adipose tissue under overnutrition, the role of IL-6 in iron metabolism has been studied more closely. In addition, the expression levels of hepcidin increased in visceral adipose tissue of HFD-induced obesity mice, in parallel with IL-6, which occurred in macrophages of adipose tissue, not in adipocytes (Gotardo et al. 2013). Another study has found that IL-6 and hepcidin are highly expressed in sperms of obese individuals, which may be associated with their high expression of miR-155 and miR-122, two microRNAs related to inflammation and iron metabolism (López et al. 2018). As an important indicator, IL-6 has been widely used in experiments to study the influence of obesity on iron metabolism, and it’s generally believed that IL-6 takes a part in iron metabolism by regulating the expression of hepcidin in obesity.

Almost in the same way as IL-6, TNFα accommodates iron homeostasis through regulating hepcidin expression. It has been shown that the increase expression of TNFα can stimulate hepcidin generation, and the application of TNFα inhibitors can reduce hepcidin production and improve anemia (Atkinson et al. 2018; Song et al. 2013). Moreover, TNFα significantly down-regulates HJV expression and inhibits liver ferritin protein production in a time-dependent manner, which is not via the BMP/SMAD signaling pathway (Salama et al. 2012). Park et al. (2017) found that the levels of iron and ferritin in the liver of C57BL/6J mice were negatively correlated with the gene level of the TNFα. It has also been considered that IL-6 is required for TNFα in the regulation of hepcidin (Nikonorov et al. 2015). Therefore, the specific mechanism of TNFα in regulating hepcidin expression and its effect on iron metabolism still needs to be further explored.

Leptin is a protein product encoded by the leptin gene (ob), which is secreted predominantly by adipose tissue and acts in regulating feeding behavior (Davis et al. 2010). In a population study, it has been found that serum leptin level in obese people is higher compared with people with normal weight, and is directly correlated with the serum hepcidin level (del Giudice et al. 2009). The correlation was still significant after BMI, gender, puberty, IL-6 and other values were adjusted. Yamamoto et al. (2018) have shown that serum hepcidin level significantly decreases in ob/ob mice with leptin deficiency and db/db mice with leptin receptor deficiency. Chung et al. (2007) found that leptin was able to induce hepatic hepcidin expression through the IL-6/STAT signaling pathway, similar to IL-6-mediated hepcidin expression. The authors observed that hepcidin mRNA expression was significantly enhanced in leptin-treated HuH7 human hepatoma cells, whereas this response was significantly attenuated after preincubation with Janus Kinase (JAK2) inhibitors. Furthermore, hepcidin promoter activity was increased in the presence of leptin, whereas this effect was reduced by mutating the STAT3 binding motif in the hepcidin promoter or co-expressing a dominant STAT3-negative mutation, suggesting the involvement of leptin in the regulation of hepcidin. Nevertheless, the recombinant leptin therapy can significantly restore the serum hepcidin level and the hepcidin mRNA expression level in the liver, implying an interaction between adipocytes and hepatocytes, which may be mediated by leptin. Matra et al. (2015) further revealed that obesity can generate a decrease of iron bioavailability, which may be attributed to the impaired iron recycling function caused by an elevated level of leptin in obesity.

The adipose tissue of obese patients can not only indirectly regulate the expression of hepcidin by secreting adipokines and proinflammatory cytokines, but also directly express hepcidin to affect iron homeostasis. In murine models fed with HFD, a significant elevated hepcidin mRNA level in visceral adipose tissue was observed (Gotardo et al. 2013). With an in vitro adipose tissue explant stimulation experiment, Bekri et al. (2006) uncovered that hepcidin was expressed not only in the liver, but also in adipose tissue at mRNA and protein levels, pointing to the potential role of adipose tissue in systemic iron homeostasis. The specific function of hepcidin expressed by adipose tissue in vivo still remains unclear. Bekri further indicated that the hepcidin mRNA level in adipose tissue of obese patients significantly increased, and this increase was associated with IL-6 and other inflammatory factors (Bekri et al. 2006). Hence, they believe that proinflammatory cytokine may be involved in the development of inflammatory hyposideremia (low serum iron levels), in obesity. This finding is of great significance for uncovering the relationship between iron metabolism disorders and obesity, especially the position of adipose tissue should not be ignored (Bekri et al. 2006). In short, the involvement of adipose tissue in iron imbalance in obese individuals deserves attention, whether through expressing hepcidin or proinflammatory cytokines/adipokines.

The discovery of obesity and iron deficiency can be traced back as far as 1962. When hyposideremia was identified in obese adolescents by Wenzel et al. (1962). Clinically, obesity treatment surgery reduces the risk of obesity-related complications, but increases the risk of nutritional complications because it artificially alters the physiological state and anatomy of the gastrointestinal tract (Lupoli et al. 2017). In a study (Coimbra et al. 2018) of 20 individuals who underwent laparoscopic adjustable gastric banding (LAGB) for 13 months, Coimbra et al. observed a significant reduction in body weight and BMI, accompanied by decreased levels of hepcidin, ferritin and inflammatory factors such as IL-6, from which they inferred that decreased levels of IL-6 may be responsible for reduced hepcidin. Iron supplementation is also an important approach to improve obesity-induced hypoferritinemia, but it does not alter obesity-induced changes in adipokines, nor does it alter iron-regulating factor levels in adipose tissue (Gotardo et al. 2016).

## Iron metabolism and NAFLD

In concert with the global epidemic of obesity, NAFLD is increasing in prevalence and accounts for approximately 25% of the world’s population (Araújo et al. 2018). More alarming is a risen diagnosed NAFLD at increasingly younger ages, and it becomes the most common chronic liver disorders in both adults and children from all ethnic backgrounds (Zhang et al. 2021). To make matters worse, both metabolically healthy and metabolically unhealthy obesity may be associated with the occurrence and development of NAFLD (Lonardo et al. 2020). In early 2020, a panel of international experts from 22 countries proposed a new definition of “metabolic dysfunction-associated fatty liver disease (MAFLD)” to replace NAFLD (Eslam et al. 2020). Based on the use of NAFLD in a large number of previous literatures, this nomenclature is still adopted in this review.

### The vital role of liver in iron absorption and utilization

Iron can enter liver cells in many forms. Once non-heme iron is ingested, Fe^3+^ is reduced by duodenal cytochrome *b* (Dcytb) and transported to the intestinal tract by DMT1. Fe^3+^ is exported by Fpn, binds to transferrin (diferric transferrin, Tf-Fe^2+^), travels to tissues, and is primarily used in the formation of new red blood cells. In addition to exogenous intake, macrophages could recycle iron from senescent red blood cells. Under the precise regulation of hepcidin, The release of iron from enterocytes, red blood cells, and macrophages is precisely controlled by Fpn and transported to various tissues and organs such as the liver (Nemeth et al. 2004). Interactions between hepatic hepcidin and Fpn as a critical mechanism for preserving iron homeostasis (Nemeth et al. 2004). In the absence of transferrin receptor (TfR) or in excess of Fe^3+^, the metal transporter SLC39A14 (solute carrier family 39 member 14) mediated the entry of NTBI into liver cells. In the recent study by Yu et al. (2020), SLC39A14 has been shown to promote iron-induced hepatocyte ferroptosis in liver-specific Trf knockout mice through its ability to transport NTBI, which is considered as a novel molecular mechanism of liver fibrosis and cirrhosis.

### Dysmetabolic iron overload syndrome in NAFLD

An increase in ferritin concentration is a key feature of iron dysregulation in NAFLD subjects. Approximately one-third of NAFLD patients had higher serum ferritin concentrations than normal. Dysmetabolic iron overload syndrome (DIOS) is characterized precisely by methemoglobinemia and is accompanied by mild iron accumulation in the hepatic reticuloendothelial cells (Aigner et al. 2008; Deugnier et al. 2017).

In NAFLD with iron overload, the iron exporter Fpn in the liver and the duodenum is lower than in normal individuals and hemochromatosis patients (Aigner et al. 2008). At the same time, duodenal iron absorption was reduced in DIOS patients (Zoller et al. 2001). Low Fpn expression is associated with insufficient dietary iron fortification in obese patients (Zimmermann et al. 2008). Phagocytosis of fragile erythrocytes by liver Kupffer cells could be another mechanism. Erythrocytosis consumes heme iron, which contributes to iron buildup in NAFLD, then promotes oxidative stress and inflammation. This was discovered in HFD-fed rabbits, as well as in vitro phagocytosis of fragile erythrocytes (Sonnweber et al. 2012).

According to a randomized cohort study, iron depletion by bloodletting did not improve metabolic and hepatic features in DIOS patients, which was related with unimproved weight gain, and was not tolerated as expected; dietary and lifestyle adjustments remain the major interventions for DIOS (Lainé et al. 2017).

### Involvement of ferroptosis in NAFLD

Ferroptosis is an iron-dependent non-apoptotic necrotic cell death form. This mechanism of cell death is specifically triggered by the depletion of cysteine, which also results in the depletion of the intracellular pool of reduced glutathione (Dixon et al. 2012). The biological features of ferroptosis, as described by Dixon, primarily include ROS buildup, iron accumulation, glutathione depletion, inhibited cystine uptake by the system Xc^−^ (cystine/glutamate antiporter), etc., accompanied by mitochondrial atrophy, mitochondrial cristae reduction or even disappearance, nuclear morphology and other morphological characteristics.

Ferritin, which stores intracellular Fe^3+^, is considered to be an important negative regulator of ferroptosis. It has been shown that the consumption of ferritin causes iron to be released into unstable iron pools, resulting in a higher sensitivity to ferroptosis. Nuclear receptor coactivator 4 (NCOA4) is a recently discovered nuclear receptor co-activator that binds to ferritin in the liver and transports it to lysosomes to release free iron, which is called ferritin-targeted autophagy (Hou et al. 2016). NCOA4 also leads to an increase in iron regulatory protein (IRP)2 and TfR1 (Mancias et al. 2014), which in turn, contributes to the accumulation of large amounts of iron that increases the susceptibility to ferroptosis.

Lipid metabolism is closely related to ferroptosis. Phosphatidylethanolamines (PEs) containing arachidonic acid (AA) or adrenaline (AdA) are key membrane phospholipids that can be oxidized to phospholipid hydroperoxides (PE-AA/AdA-OOH) via non-enzymatic reactions, thereby driving ferroptosis (Dixon et al. 2015). Loguercio et al. showed elevated levels of malondialdehyde (MDA) and 4-hydroxynonenal (both markers of lipid peroxidation) in the vast majority of NAFLD patients, a phenomenon that was particularly pronounced in nonalcoholic steatohepatitis (NASH) patients (Loguercio et al. 2001). Moreover, the accumulation of lipid peroxides and ROS promoted ferroptosis in the liver of methionine/choline deficiency diet (MCD)-fed mice, and the concurrent use of ferroptosis inhibitors alleviated MCD diet-induced inflammation and liver fibrosis (Li et al. 2020). Similarly, In another NASH mouse modeling by MCD feeding, liver steatosis aggravated after RSL3 (ferroptosis inducer) administration, whereas the severity of NASH was significantly alleviated after treatment with sodium selenite (GPX4 activator), deferoxamine (iron-chelating agent) and ferrostatin-1 (ferroptosis inhibitor) (Qi et al. 2020). Furthermore, in a choline-deficient and ethionine-supplemented diet-induced NASH model, ferroptosis was found to be a preemptive event leading to NASH and preceding other types of cell death (Tsurusaki et al. 2019) (Fig. 2 ②).

## Iron metabolism and diabetes

Diabetes is a chronic disease characterized by hyperglycemia, in which the body is unable to produce enough insulin or the action of insulin is impaired (Sims et al. 2021). As the increasing number of people with diabetes, it ranks just behind tumors and cardiovascular diseases among chronic diseases in terms of risk to human health (Collaboration 2016). An estimated of 537 million adults (aged 20–79) are diabetic worldwide, accounting for 10% of the world’s population in this age group, according to the latest Diabetes Atlas 2021 report from the International Diabetes Federation (IDF). By 2030 and 2045, the figures are respectively expected to reach 643 million and 783 million (IDF 2021).

### Iron overload and pancreatic oxidative stress

Tuomainen et al. found a significant correlation between increased body iron storage and elevated blood glucose in a cohort study as early as 1997 (Tuomainen et al. 1997). After that, Ford et al. proposed the hypothesis that elevated serum ferritin was associated with an increased risk of diabetes in a population study in 1999 (Ford and Cogswell 1999), which has been confirmed by subsequent studies (Huth et al. 2015; Podmore et al. 2016). Previous studies have verified that iron metabolism disorder is one of the risk factors of diabetes (Kataria et al. 2018), and researchers further implied the close correlation between iron dyshomeostasis and gestational diabetes mellitus (Kataria et al. 2018; Wang et al. 2015). Most studies have inclined to identify iron overload as a risk factor for T2DM (Aregbesola et al. 2013; Montonen et al. 2012; Sun et al. 2013). Excessive iron in the pancreas may lead to defects in the synthesis and secretion of insulin, while improving pancreatic iron overload can reduce oxidative stress and ameliorate diabetic complications (Minamiyama et al. 2010). Although the exact mechanism of iron metabolic disturbance in the development of diabetes is not well understood, oxidative stress is thought to be one of the core mechanisms that correlate excess iron with a higher incidence of T2DM via mediating several key events such as insulin resistance and β cells dysfunction (Horinouchi et al. 2019). Pancreatic β-cells are rich in highly active mitochondria and are highly sensitive to ROS (Shirasuga et al. 1989). Further correlation analysis showed that superoxide dismutase (SOD), MDA, glutathione (GSH), and GPX were all correlated to Tf and TfR levels, among which MDA and GPX were correlated with iron content, indicating that iron metabolism disorder is involved in the process of oxidative stress (Bao et al. 2012; Huth et al. 2015). The study by Blesia et al. has shown that exposure to high iron (100 µM) results in cellular oxidative damage and initiates insulin secretory dysfunction in pancreatic β-cells by reducing the expression of synaptosomal associated protein 25, a key protein involved in the insulin exocytosis machinery (Blesia et al. 2021). It is noteworthy that other more severe iron overload models affecting hepcidin expression, such as the hepcidin-resistant model bearing the p.C326S mutation in Fpn, as well as the Hampsand HJV knockout model, do not show liver disease or endocrine problems despite pancreatic iron accumulation, probably due to greater resistance to oxidative stress injury of these mouse models (Altamura et al. 2020). The pancreatic and duodenal homeobox 1 (PDX1) and V-Maf avian musculoaponeurotic fibrosarcoma oncogene homolog A (MafA), two critical transcription factors involved in the control of insulin gene expression, are both targets for ROS (Cnop et al. 2014), and decreased hepcidin expression in MIN6 cells leads to inhibited insulin synthesis via iron overload and decreased PDX1 expression (Mao et al. 2017; Shu et al. 2019).

The chronic hyperglycemia status will increase the expression of stress genes, including HO-1 gene (Li et al. 2015). The human HO-1 gene has a GT site in the proximal promoter region as well as transcription factor nuclear factor κB (NF-κB) and activator protein (AP-2) site (Chau 2015). In T2DM, the HO-1 gene is highly active and exhibits the characteristic of polymorphic gene promoters in its gene sequence, which leads to an increase in iron storage in the body. This is mainly due to the active participation of HO-1 in the degradation of haemachrome, while iron released during the degradation process is potentially cytotoxic (Ryter 2021). Therefore, an increase in the expression of HO-1 gene causes iron overload in the body to promote the occurrence of oxidative stress and stimulate the development of T2DM. As mentioned above, as a strong oxidizing agent, iron can promote the occurrence of oxidative stress, and excessive oxidative stress is one of the precursors of T2DM (Jin et al. 2013). Thus, hyperglycemia increases HO-1 gene expression and exacerbates iron overload, and vice versa.

### Iron overload and Adenosine 5′-monophosphate-activated protein kinase (AMPK) activity

AMPK is a negative feedback regulator of insulin secretion in β-cells, and insulin secretion will be inhibited when the activity of AMPK increases. Consequently, when the concentration of serum glucose drops, the gene expression of active AMPK will be increased, thereby inhibiting insulin secretion (Hardie 2013); while the activity of AMPK is gradually reduced with the increase of glucose concentration. However, in skeletal muscle and liver, the activated AMPK can overcome a reduction in insulin secretion arising from the activation of AMPK in β-cells induced by HFD, and ultimately leading to an increase in glucose tolerance by stimulating glucose uptake and fatty acid oxidation in peripheral tissues, and inhibiting gluconeogenesis in the liver (Huang et al. 2011). In the mice model of hemochromatosis established by Huang et al., iron overload can reduce the oxidation of glucose and induce the phosphorylation of AMPK in skeletal muscle. Isolated soleus muscle from hemochromatosis mice demonstrated an absolute increase in the capacity for fatty acid oxidation compared with wild type, which was consistent with AMPK role in regulating fatty acid oxidation (Huang et al. 2011). The difference in functions between AMPK in β-cells and skeletal muscle and liver might be caused by the expression of different subtypes of AMPK in peripheral tissues of insulin production (β-cells) and insulin response (Kjøbsted et al. 2016).

### Iron overload and pancreatic islet amylin

Another mechanism by which iron overload may affect β-cells function and survival is via amylin. Misfolding and aggregate deposition of human islet amyloid polypeptide (hIAPP) in the extracellular matrix and within β-cells have been detected post-mortem in the pancreas of 90% of subjects affected by T2DM (Clark et al. 1988; Röcken et al. 1992), where the polypeptide shows cytotoxic activity caused by the disruption of the cell membrane, perturbed ion homeostasis, endoplasmic reticulum stress, mitochondrial damage and final oxidative stress (Bishoyi et al. 2021). Intriguingly, iron has been shown to enhance amylin fB-sheet formation, triggering their aggregate deposition (Alghrably et al. 2019). Furthermore, heme can also bind to amylin to form a complex that leads to H_2_O_2_ formation via oxidative stress (Mukherjee and Dey 2013), thus fostering ROS-mediated β-cells failure (Fig. 2 ③).

## Iron metabolism and coronary atherosclerotic heart disease (CAD)

CAD is the leading cause of death in both developing and developed countries, according to the World Health Organization, with an estimated 23.6 million deaths per year by 2030. CAD is caused by atherosclerotic lesions in the coronary arteries that narrow or block the lumen of the vessels, resulting in ischemia, hypoxia or necrosis of the myocardium, often referred to as “coronary heart disease”. Risk factors for cardiovascular disease such as elevated cholesterol and hypertension are highly prevalent among overweight and obese individuals (La Sala and Pontiroli 2020). It has been suggested that children and adolescents with severe obesity have a significant incidence of cardiovascular disease and a higher risk of all-cause mortality compared to those with mild obesity (Bendor et al. 2020).

Iron enters cardiomyocytes via the TfR1, as well as additional channels such as the T-type calcium channel (TTCC), DMT1, L-type voltage-dependent Ca^2+^ channel (LTCC), Zinc–Iron regulatory protein (ZIP)8, and 14. The Fpn is the only protein that extrudes iron from heart muscle cells. Specially, H-ferritin-like protein is present in cardiac mitochondria for iron storage. Mitochondrial ferritin (mtFT) has been shown to protect cardiomyocytes from oxidative stress induced by cardiac injury by increasing mitochondrial sensitivity (Li et al. 2017). Besides, IRP is responsible for modulating iron homeostasis in cardiomyocytes. When myocardial iron level is low, IRP expression is increased to reduce Fpn and ferritin expression, in turn inhibiting iron export and storage, and to boost TfR1 expression, so increasing cell iron availability.

Both iron deficiency and iron overload are associated with cardiovascular disease risk (Basuli et al. 2014; Lapice et al. 2013). As early as forty years ago, Sullivan proposed that the risk of cardiovascular disease was positively correlated with iron accumulation in atherosclerosis (Sullivan 1981). According to Vinchi’s latest research, iron heavily deposited in arterial mediums is associated with plaque formation, vascular oxidative stress and dysfunction (Vinchi et al. 2020). NTBI induces iron overload in cultured vascular cells, which leads to ROS production and apoptosis, and stimulates abundant monocyte chemotactic protein (MCP)1-mediated monocyte aggregation, a potential intrinsic trigger of atherosclerosis (Vinchi et al. 2020). A new report found that the Sirtuin-1 (SIRT1), an autophagy agent in foam cells, could block ferroptosis incited by excessive iron (Su et al. 2021). However, superfluous iron restrains the SIRT1-autophagy pivot of froth cells and the activity of GPX4, which together incite ferroptosis of foam cells (Su et al. 2021). By adding the exogenous ox-LDL and ferric ammonium citrate to THP-1 cells, Su et al. affirmed that unnecessary iron prompted ferroptosis in froth cells. Although the mechanism by which iron overload leads to coronary atherosclerosis is not fully understood, restricting dietary iron or using iron chelation therapy is supposed to mitigate atherosclerosis (Su et al. 2021). Therefore, the overwhelming evidence supports a link between iron overload and CAD. In addition, coronary artery calcification is an independent risk factor for CAD and is closely associated with plaque rupture, while ferroptosis can also lead to CAD by promoting vascular calcification (Durham et al. 2018).

The iron hypothesis remains controversial because the mechanism by which iron accumulation causes CAD is not fully understood. Omar Saeed et al. have uncovered that pharmacological inhibition of hepcidin in C57BL/6J mice (increased iron in tissues) was able to increase cholesterol efflux from macrophages, reduce foam cell formation, and ultimately slow down the process of atherosclerosis (Saeed et al. 2012). An epidemiological study using a Mendelian randomization approach supports the notion that higher iron levels can reduce the risk of CAD (Gill et al. 2017). Surprisingly, in a recent advance in iron metabolism research, Léon Kautz’s team found no direct correlation between iron levels in mice and the risk of atherosclerosis (Kautz et al. 2013) (Fig. 2 ④).

## Iron metabolism and cardiomyopathy

Obesity cardiomyopathy is defined as myocardial changes associated with obesity, independent of other heart diseases or risk factors (Wong and Marwick 2007). Originally, obese cardiomyopathy was defined as heart failure primarily caused by obesity and was thought to be confined to severely obese individuals. However, this definition was later extended to include cardiomyopathies in obese that could not be explained by other etiologies such as hypertension, diabetes mellitus, or CAD (Wong and Marwick 2007). Thus, obesity as an important risk factor for the development of heart failure has been identified (Hao et al. 2019; Kenchaiah et al. 2002). This was in response to a growing body of evidence highlighting myocardial alterations in people with mild to moderate obesity. Cardiomyocytes are terminally differentiated cells, which can’t be regenerated. Once dead, they can only be replaced by scar tissue, leading to structural and functional impairment of the heart and eventually to the development of heart failure (Smits et al. 2018).

### Iron overload and cardiomyopathy

Likewise, iron overload may also damage the heart, leading to cardiomyopathy. As mentioned earlier, excessive iron acts as a catalyst in Fenton reaction to induce oxidative stress and lead to ROS accumulation, which interferes with Ca^2+^ homeostasis in cardiomyocytes and multiple ion transporters responsible for myocardial electrical activity, sequentially leading to cardiac diastolic and systolic dysfunction and arrhythmia (Kawabata 2019). The increase in mitochondrial ROS production also leads to the depolarization of mitochondrial membrane potential and the opening of mitochondrial permeability transition pore, which causes cell rupture and eventually leads to cardiac dysfunction and cardiomyopathy (Kumfu et al. 2012). Furthermore, the accumulation of iron in the heart has been supposed to depend on the penetration of ferrous iron (Fe^2+^) through the L-type voltage-dependent Ca^2+^ channel (LVDCC), and the transgenic mice with iron overload and heart-specific overexpression of LVDCC α1 subunit exhibited higher levels of myocardial molten iron and oxidative stress, leading to more severe impairment of heart function; while inhibition of LVDCC α1 had a protective effect on myocardium (Oudit et al. 2003).

### Ferroptosis and myocardial injury

Wang et al. have demonstrated that the expression of mixed lineage kinase (MLK)3, a member of MAP3K family, is significantly increased in the model of myocardial hypertrophy induced by pressure load in mice. A subsequent study has indicated that MLK3 not only regulates the inflammatory response induced by pyroptosis through NF-κB/NLRP3 signaling pathway, but also modulates ferroptosis induced by oxidative stress through JNK/p53 signaling pathway. Both of these biological events aggravate myocardial hypertrophy and fibrosis, while miR-351 plays a protective role in myocardial hypertrophy by directly acting on MLK3 to inhibit the occurrence of pyroptosis and ferroptosis (Wang et al. 2020). Beclin1, also known as BECN, is the homologous gene of yeast autophagy gene Atg6/Vps30, which is an essential molecule in the process of autophagy. Yin et al. discovered that mice with insufficient single gene dosage of Beclin1 could withstand the myocardial hypertrophy induced by low temperature. With further experiments in vivo and in vitro, it has turned out that insufficient single gene dosage of Beclin1 raises the level of solute carrier family 7 member 11(SLC7A11) and GPX4, and lowers the level of NCOA4, a ferritin degradation autophagic cargo receptor. The increased NCOA4, which is closely related to autophagy, can aggravate iron accumulation and lipid peroxidation, thus promoting the occurrence of ferroptosis and exacerbating myocardial hypertrophy (Yin et al. 2020). Thereby, these two studies have revealed novel mediating actions of MLK3 and Beclin1 between ferroptosis and myocardial injury.

In mice with heart-specific ferritin heavy chain (FTH) gene knockout, decreased iron level and enhanced oxidative stress in the heart tissue were observed, which lead to mild cardiac injury after aging; while further high-iron diet would cause hypertrophic cardiomyopathy, and ferroptosis was involved in the pathophysiological process of the disease. However, over-expression of SLC7A11 in cardiomyocytes may inhibit ferroptosis of the myocardium (Fang et al. 2020). Collectively, inhibition of ferroptosis provides a new intervention target for the prevention and treatment of cardiomyopathy (Fig. 2 ④).

## Conclusion

At present, iron metabolism has been extensively studied in obesity-related metabolic disorders, while ferroptosis is still in its infancy. Ferroptosis is a unique cell death pathway that is iron-dependent, non-apoptotic, non-necrotic and non-autophagic (Bogdan et al. 2016). Although much progress has been made in recent years in the study of ferroptosis in the above-mentioned diseases, many questions remain to be addressed. At present, it is not clear whether iron metabolism will affect lipid metabolism. What are the mechanisms of systemic and intracellular iron homeostasis crosstalk? These diseases share upstream factors, but what are the downstream pathways? These may be new research directions in the future.

In view of the great role of iron metabolism in metabolic diseases, in-depth studies of the underlying mechanism are of considerable significance for the prevention, diagnosis, treatment, and prognosis of obesity and its complications, and can provide medical practitioners with more clinical ideas.

## Data Availability

Not applicable.

## Abbreviations

DMT1: Divalent metal transporter 1
Fpn: Ferroportin
ROS: Reactive oxygen species
T2DM: Type 2 diabetes
NAFLD: Nonalcoholic fatty liver disease
CREB: CAMP-responsive element binding protein
NTBI: Non-transferrin-bound iron
ERE: Estrogen response element
HJV: Hemojuvelin
STAT: Signal transducers and activators of transcription
Hamp1: Hepcidin-1
Hamp2: Hepcidin-2
LPS: Lipopolysaccharide
BMI: Body mass index
HFD: High fat diet
IL: Interleukin
HO: Heme oxygenase
TNF: Tumor necrosis factor
RGMc: Repulsive guidance molecule C
BMP: Bone morphogenetic protein
SMAD: Drosophila mothers against decapentaplegic
JAK: Janus Kinase
LAGB: Laparoscopic adjustable gastric banding
MAFLD: Metabolic dysfunction-associated fatty liver disease
Dcytb: Duodenal cytochrome *b*
TfR: Transferrin receptor
SLC39A14: Solute carrier family 39 member 40
DIOS: Dysmetabolic iron overload syndrome
NCOA4: Nuclear receptor coactivator 4
IRP: Iron regulatory protein
PEs: Phosphatidylethanolamines
AA: Arachidonic acid
AdA: Adrenaline
MDA: Malondialdehyde
NASH: Nonalcoholic steatohepatitis
MCD: Methionine/choline deficiency diet
GPX: Glutathione peroxidase
IDF: International Diabetes Federation
GSH: Glutathione
SOD: Superoxide dismutase
PDX1: Pancreatic and duodenal homeobox 1
MafA: V-Maf avian musculoaponeurotic fibrosarcoma oncogene homolog A
NF-κB: Nuclear factor κB
AP-2: Activator protein
AMPK: Adenosine 5′-monophosphate (AMP)-activated protein kinase
hIAPP: Human islet amyloid polypeptide
CAD: Coronary atherosclerotic heart disease
TTCC: T-type calcium channel
ZIP: Zinc-Iron regulatory protein
mtFT: Mitochondrial ferritin
MCP: Monocyte chemotactic protein
SIRT1: Sirtuin-1
LVDCC/LTCC: L-type voltage-dependent Ca^2+^ channel
MLK: Mixed lineage kinase
SLC7A11: Solute carrier family 7 member 11
FTH: Ferritin heavy chain
